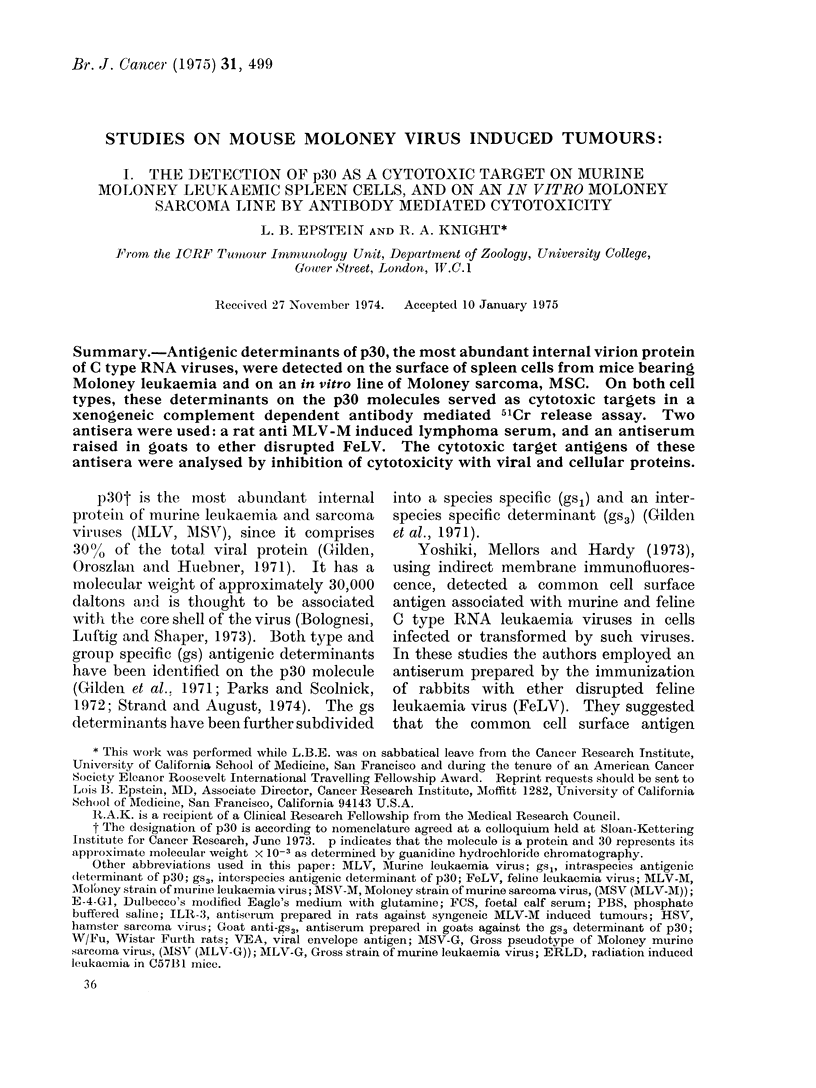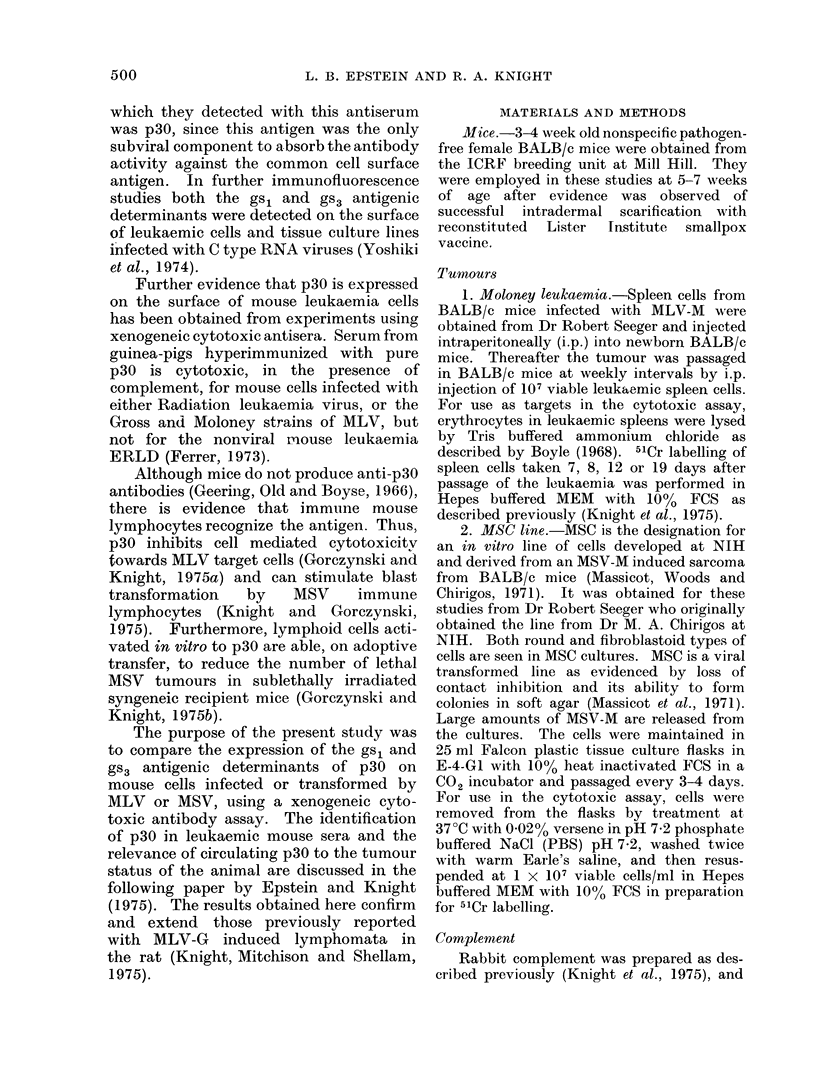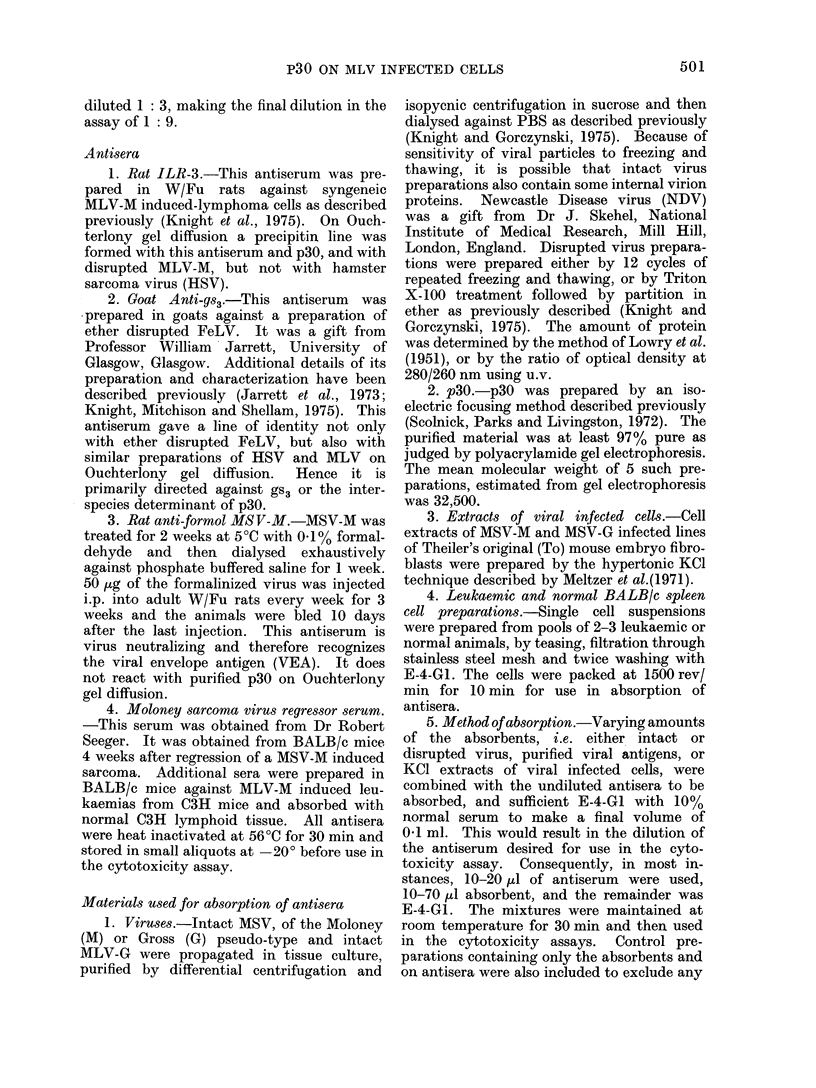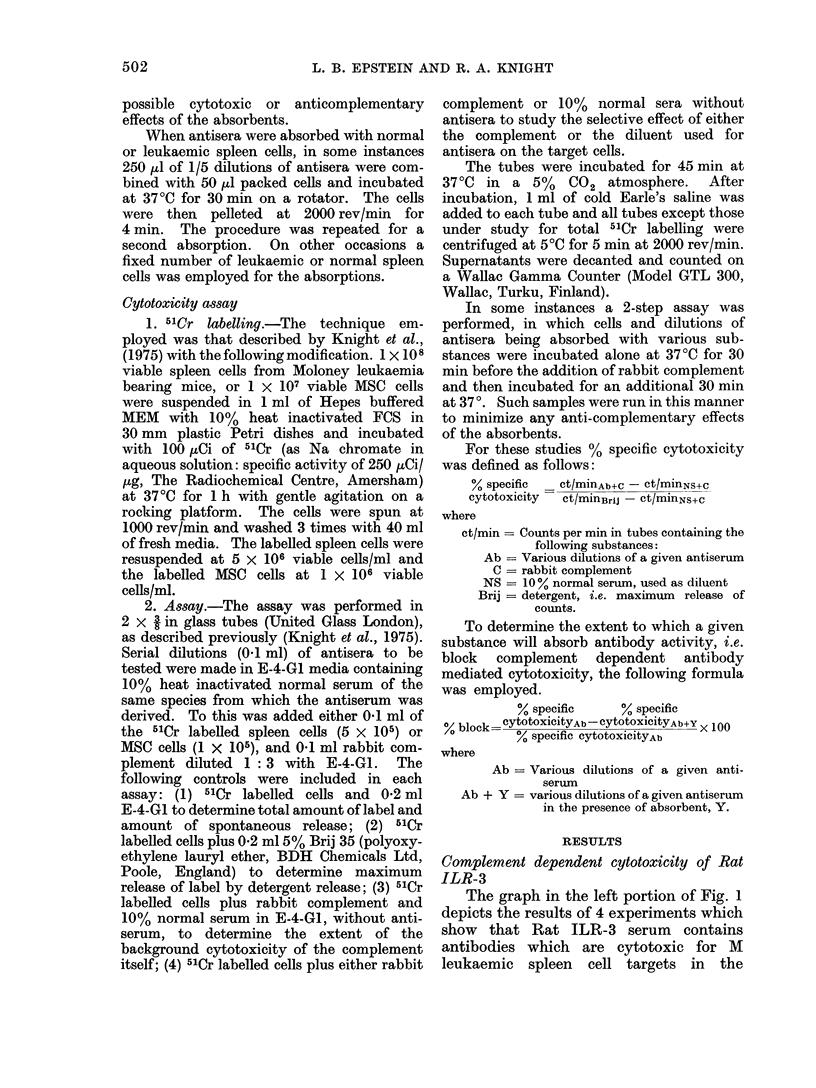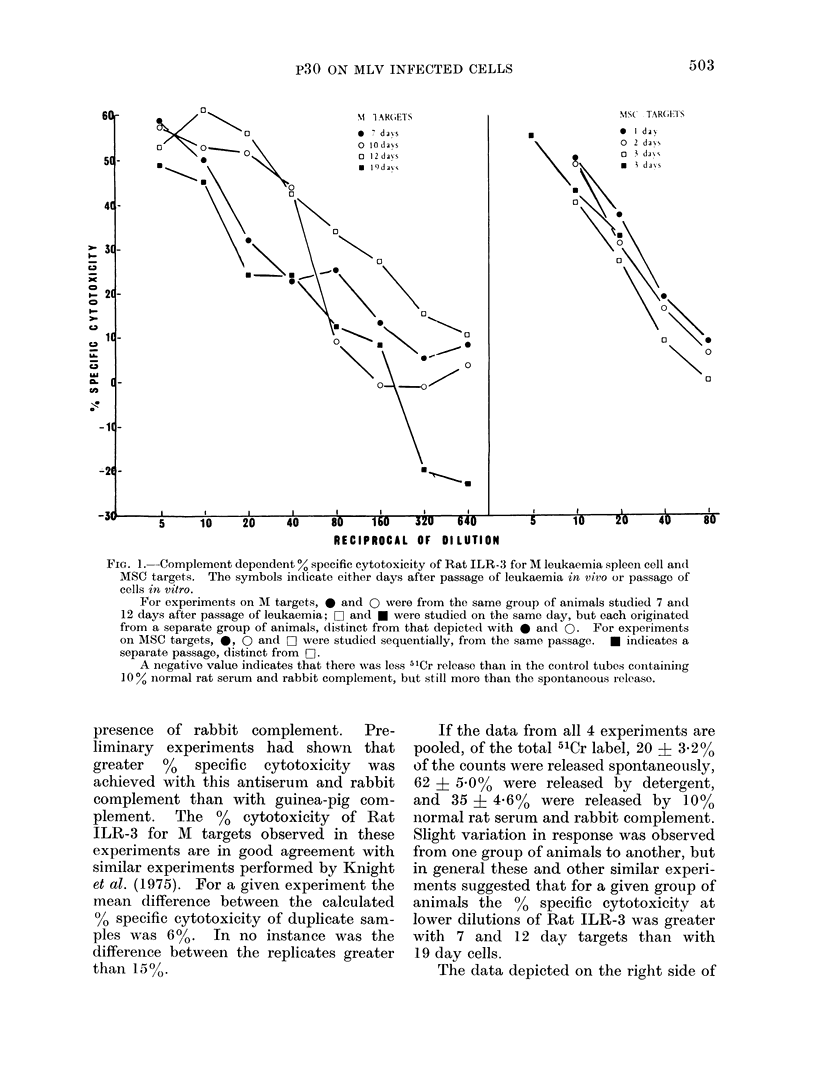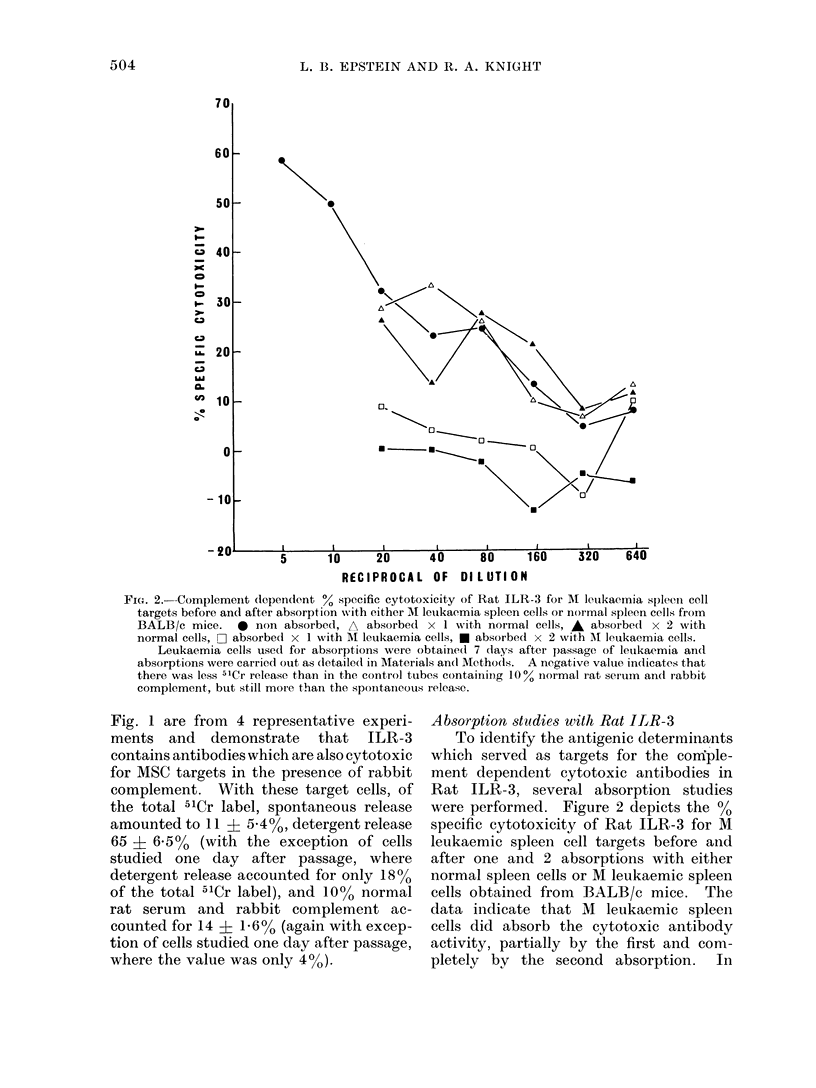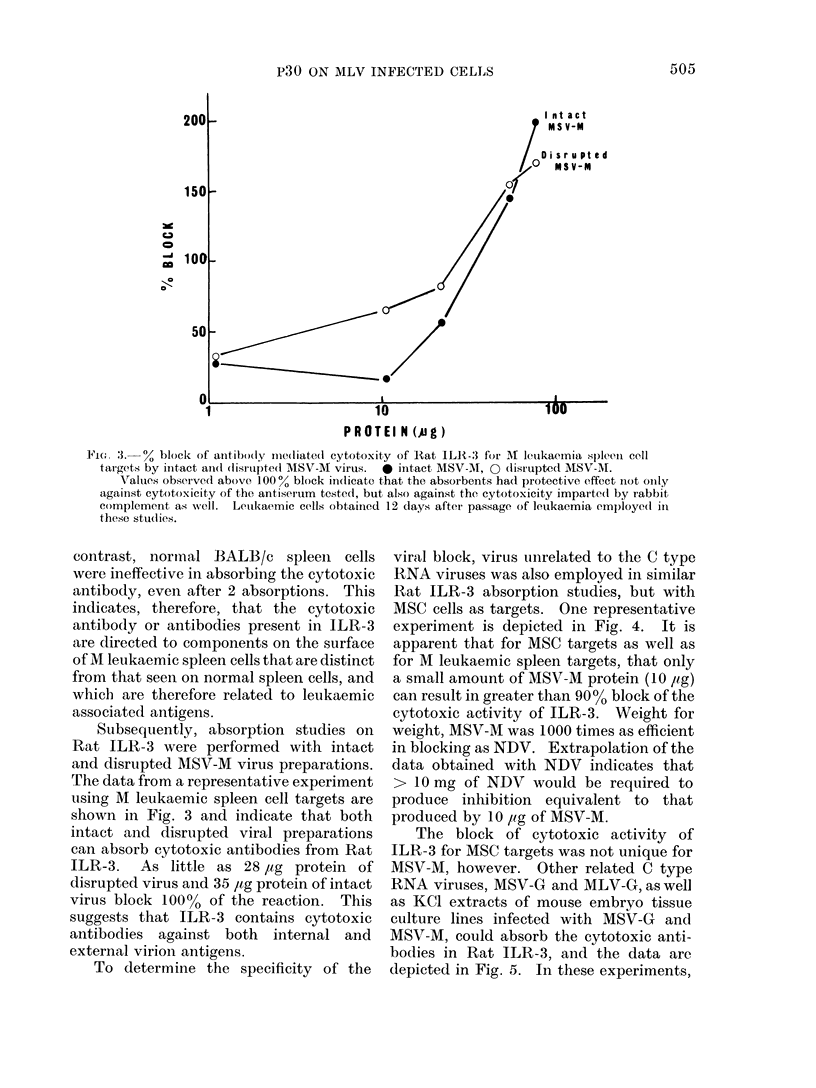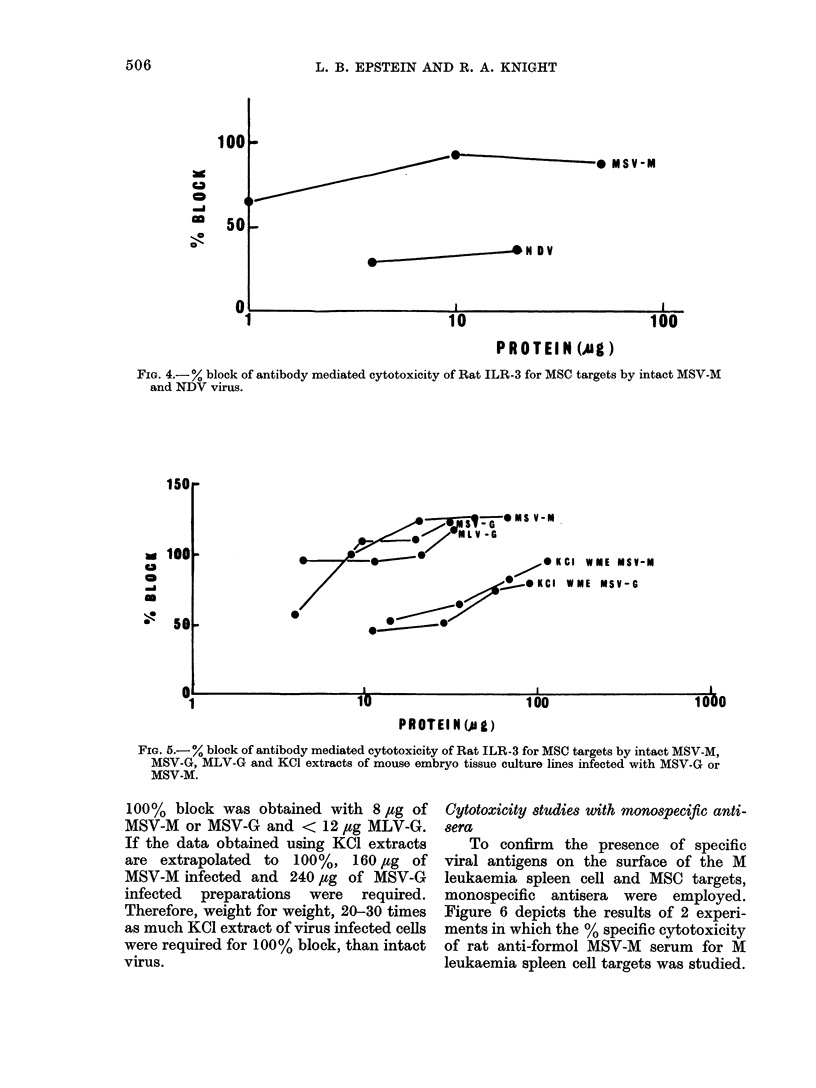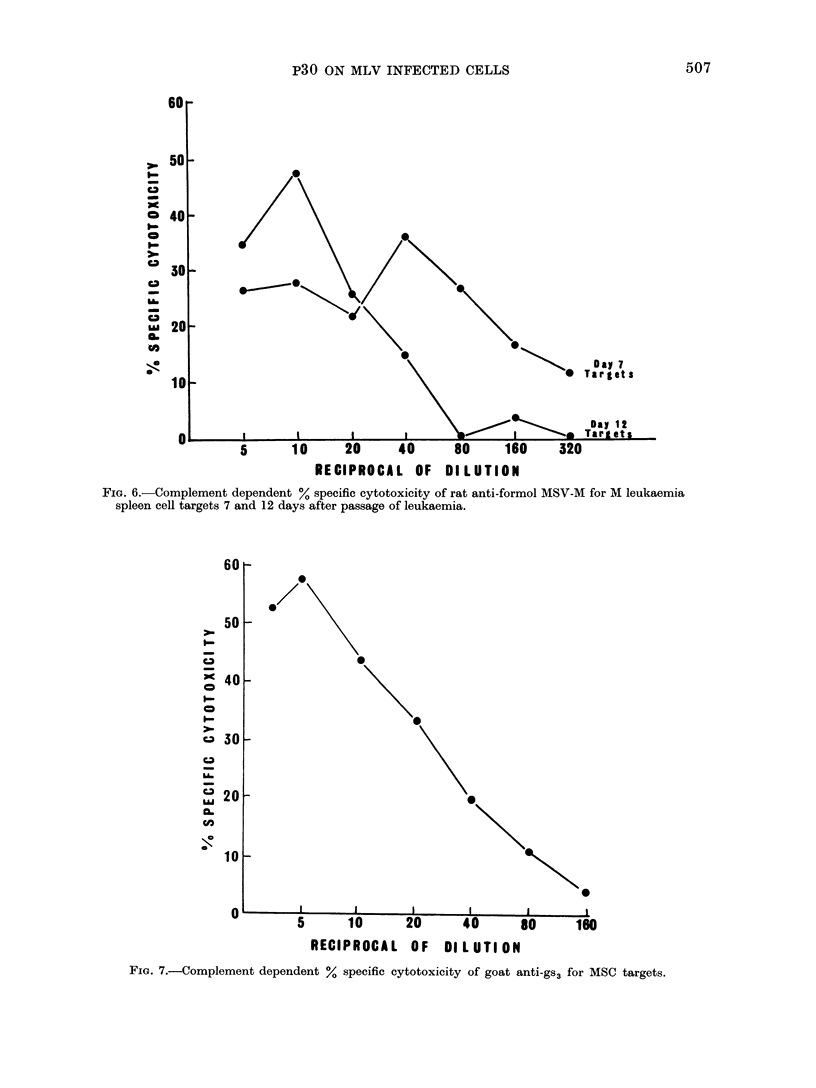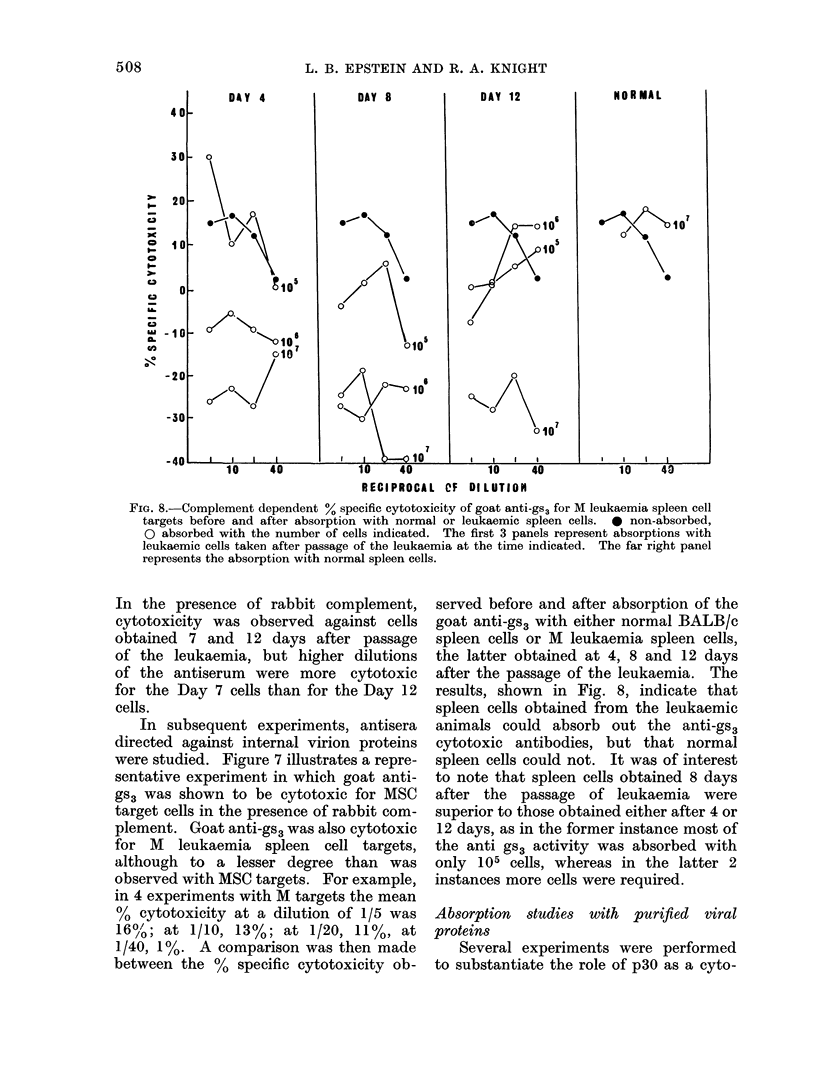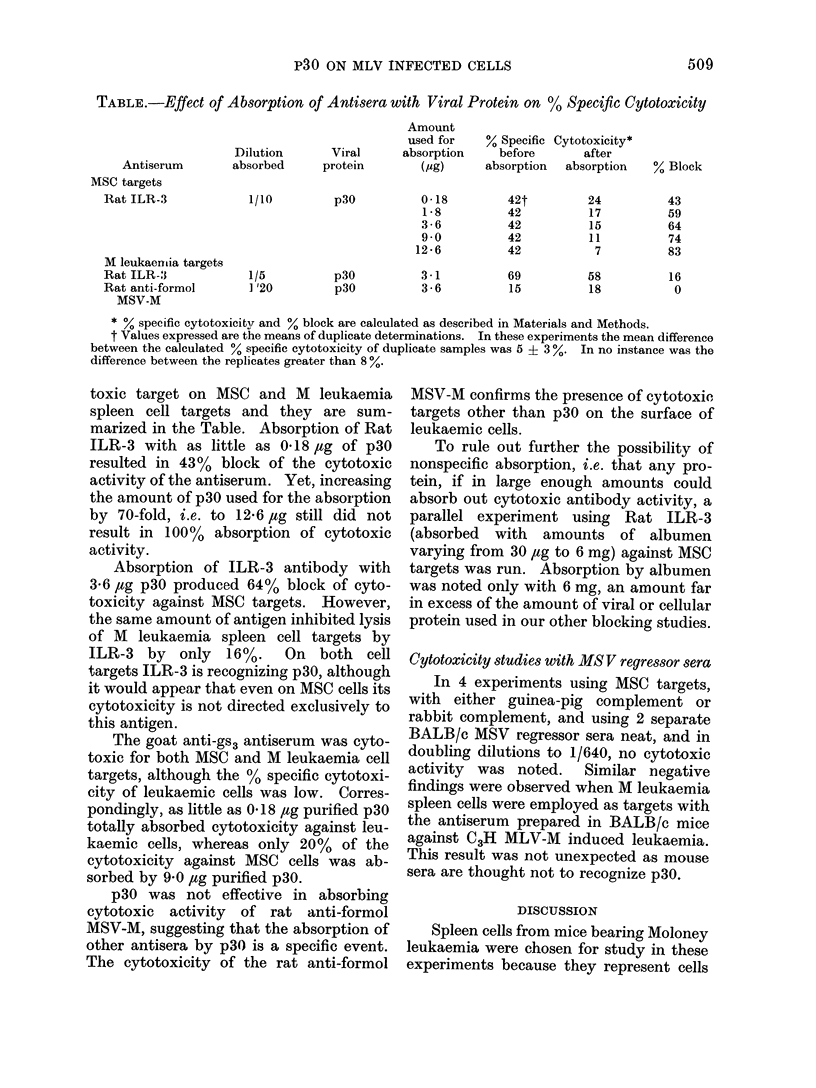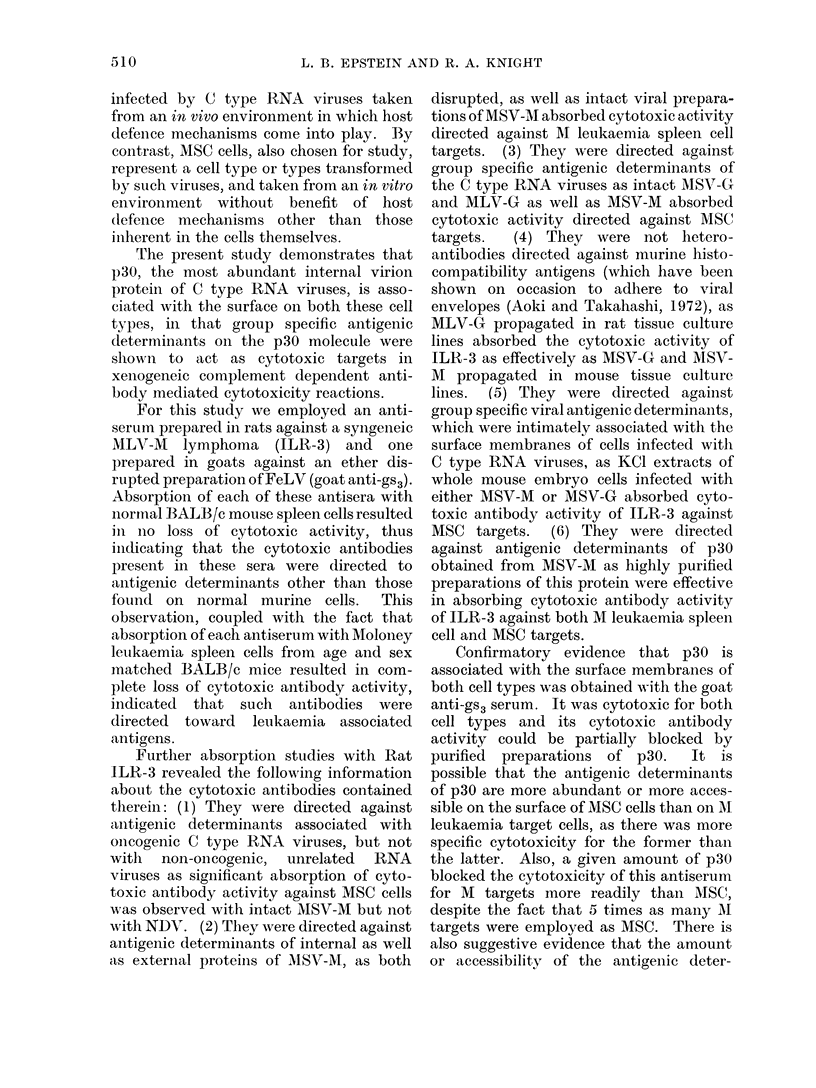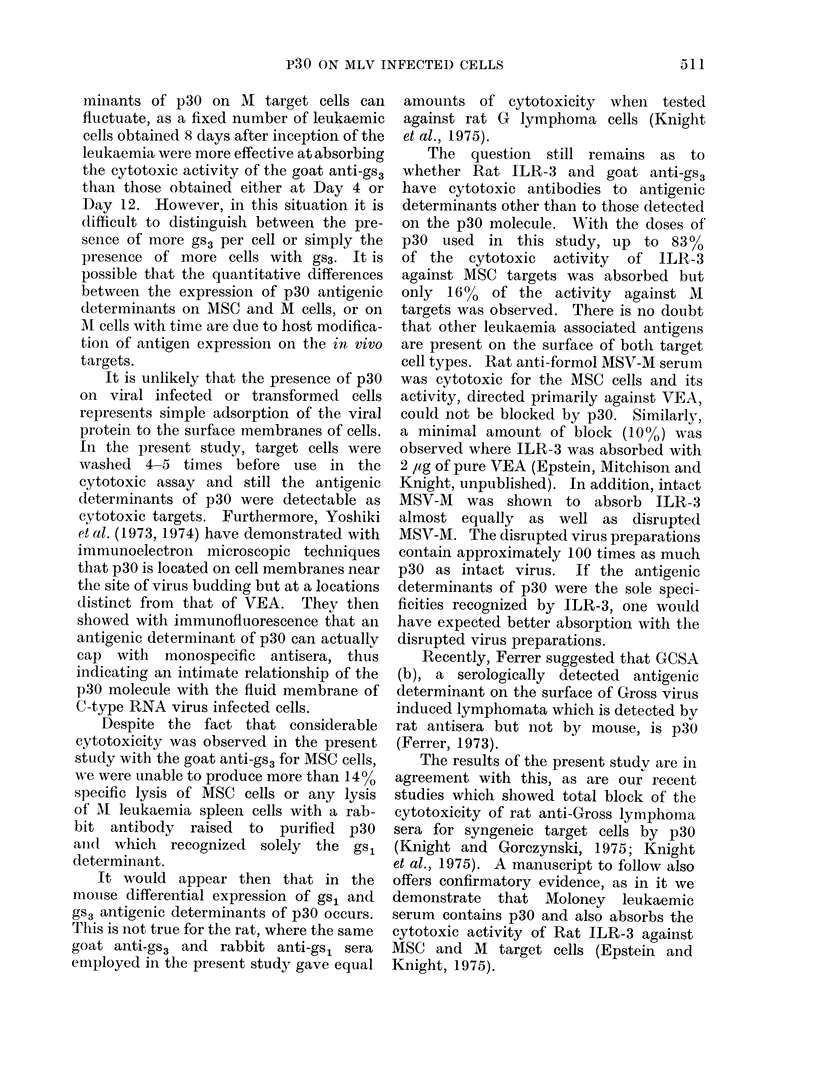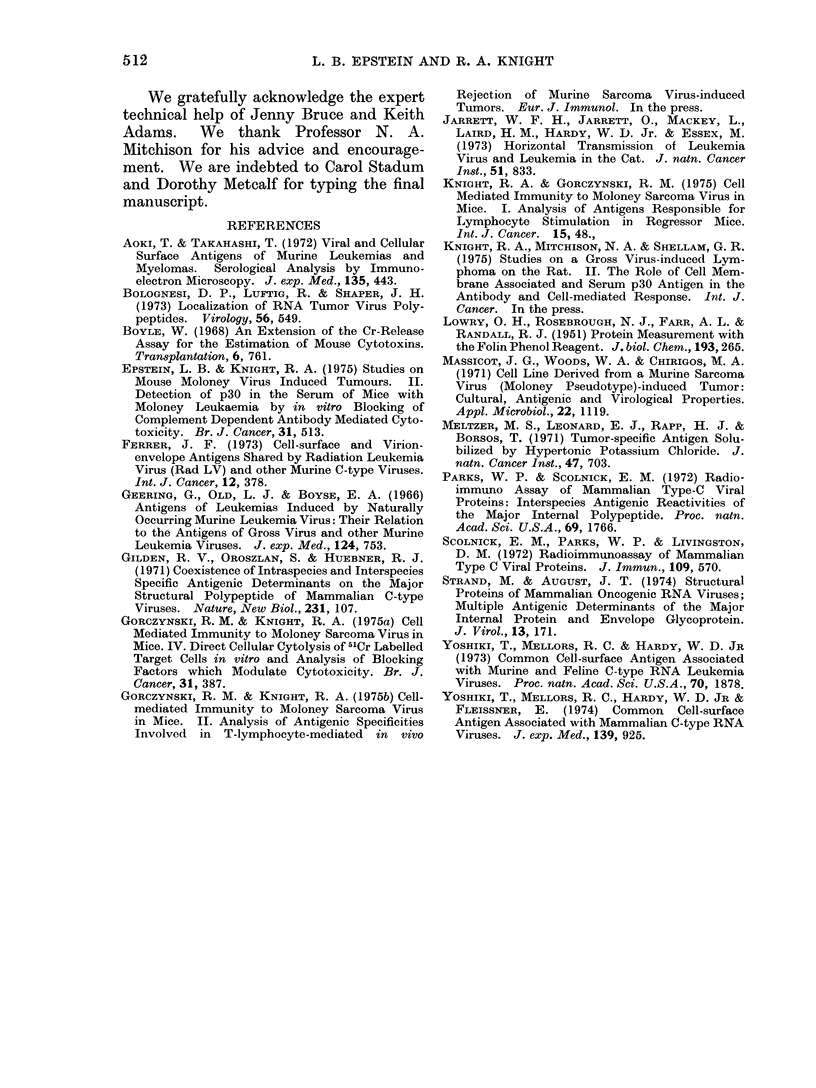# Studies on mouse Moloney virus induced tumours: I. The detection of p30 as a cytotoxic target on murine Moloney leukaemic spleen cells, and on an in vitro Moloney sarcoma line by antibody mediated cytotoxicity.

**DOI:** 10.1038/bjc.1975.90

**Published:** 1975-05

**Authors:** L. B. Epstein, R. A. Knight

## Abstract

Antigenic determinants of p30, the most abundant internal virion protein of C type RNA viruses, were detected on the surface of spleen cells from mice bearing Moloney leukaemia and on an in vitro line of Moloney sarcoma, MSC. On both cell types, these determinants on the p30 molecules served as cytotoxic targets in a xenogenic complement dependent antibody mediated 51Cr release assay. Two antisera were used: a rat anti MLV -M induced lymphoma serum, and an antiserum raised in goats to either disrupted FeLV. The cytotoxic target antigens of these antisera were analysed by inhibition of cytotoxicity with viral and cellular proteins.


*Br. J. Cancer* (1975) **31**, 499

### STUDIES ON MOUSE MOLONEY VIRUS INDUCED TUMOURS:

### I. THE DETECTION OF p30 AS A CYTOTOXIC TARGET ON MURINE MOLONEY LEUKAEMIC SPLEEN CELLS, AND ON AN *IN VITRO* MOLONEY SARCOMA LINE BY ANTIBODY MEDIATED CYTOTOXICITY

L. B. EPSTEIN AND R. A. KNIGHT*

*From the ICRF Tumour Immunology Unit, Department of Zoology, University College, Gower Street, London, W.C.*1

Received 27 November 1974. Accepted 10 January 1975

**Summary.—Antigenic determinants of p30, the most abundant internal virion protein of C type RNA viruses, were detected on the surface of spleen cells from mice bearing Moloney leukaemia and on an *in vitro* line of Moloney sarcoma, MSC. On both cell types, these determinants on the p30 molecules served as cytotoxic targets in a xenogeneic complement dependent antibody mediated $^{51}Cr$ release assay. Two antisera were used: a rat anti MLV-M induced lymphoma serum, and an antiserum raised in goats to ether disrupted FeLV. The cytotoxic target antigens of these antisera were analysed by inhibition of cytotoxicity with viral and cellular proteins.**

p30† is the most abundant internal protein of murine leukaemia and sarcoma viruses (MLV, MSV), since it comprises 30% of the total viral protein (Gilden, Oroszlan and Huebner, 1971). It has a molecular weight of approximately 30,000 daltons and is thought to be associated with the core shell of the virus (Bolognesi, Luftig and Shaper, 1973). Both type and group specific (gs) antigenic determinants have been identified on the p30 molecule (Gilden *et al.*, 1971; Parks and Scolnick, 1972; Strand and August, 1974). The gs determinants have been further subdivided into a species specific ($gs_1$) and an interspecies specific determinant ($gs_3$) (Gilden *et al.*, 1971).

Yoshiki, Mellors and Hardy (1973), using indirect membrane immunofluorescence, detected a common cell surface antigen associated with murine and feline C type RNA leukaemia viruses in cells infected or transformed by such viruses. In these studies the authors employed an antiserum prepared by the immunization of rabbits with ether disrupted feline leukaemia virus (FeLV). They suggested that the common cell surface antigen

* This work was performed while L.B.E. was on sabbatical leave from the Cancer Research Institute, University of California School of Medicine, San Francisco and during the tenure of an American Cancer Society Eleanor Roosevelt International Travelling Fellowship Award. Reprint requests should be sent to Lois B. Epstein, MD, Associate Director, Cancer Research Institute, Moffitt 1282, University of California School of Medicine, San Francisco, California 94143 U.S.A.

R.A.K. is a recipient of a Clinical Research Fellowship from the Medical Research Council.

† The designation of p30 is according to nomenclature agreed at a colloquium held at Sloan-Kettering Institute for Cancer Research, June 1973. p indicates that the molecule is a protein and 30 represents its approximate molecular weight $\times 10^{-3}$ as determined by guanidine hydrochloride chromatography.

Other abbreviations used in this paper: MLV, Murine leukaemia virus; $gs_1$, intraspecies antigenic determinant of p30; $gs_3$, interspecies antigenic determinant of p30; FeLV, feline leukaemia virus; MLV-M, Moloney strain of murine leukaemia virus; MSV-M, Moloney strain of murine sarcoma virus, (MSV (MLV-M)); E-4-G1, Dulbecco's modified Eagle's medium with glutamine; FCS, foetal calf serum; PBS, phosphate buffered saline; ILR-3, antiserum prepared in rats against syngeneic MLV-M induced tumours; HSV, hamster sarcoma virus; Goat anti-$gs_3$, antiserum prepared in goats against the $gs_3$ determinant of p30; W/Fu, Wistar Furth rats; VEA, viral envelope antigen; MSV-G, Gross pseudotype of Moloney murine sarcoma virus, (MSV (MLV-G)); MLV-G, Gross strain of murine leukaemia virus; ERLD, radiation induced leukaemia in C57Bl mice.

which they detected with this antiserum was p30, since this antigen was the only subviral component to absorb the antibody activity against the common cell surface antigen. In further immunofluorescence studies both the $gs_1$ and $gs_3$ antigenic determinants were detected on the surface of leukaemic cells and tissue culture lines infected with C type RNA viruses (Yoshiki *et al.*, 1974).

Further evidence that p30 is expressed on the surface of mouse leukaemia cells has been obtained from experiments using xenogeneic cytotoxic antisera. Serum from guinea-pigs hyperimmunized with pure p30 is cytotoxic, in the presence of complement, for mouse cells infected with either Radiation leukaemia virus, or the Gross and Moloney strains of MLV, but not for the nonviral mouse leukaemia ERLD (Ferrer, 1973).

Although mice do not produce anti-p30 antibodies (Geering, Old and Boyse, 1966), there is evidence that immune mouse lymphocytes recognize the antigen. Thus, p30 inhibits cell mediated cytotoxicity towards MLV target cells (Gorczynski and Knight, 1975*a*) and can stimulate blast transformation by MSV immune lymphocytes (Knight and Gorczynski, 1975). Furthermore, lymphoid cells activated *in vitro* to p30 are able, on adoptive transfer, to reduce the number of lethal MSV tumours in sublethally irradiated syngeneic recipient mice (Gorczynski and Knight, 1975*b*).

The purpose of the present study was to compare the expression of the $gs_1$ and $gs_3$ antigenic determinants of p30 on mouse cells infected or transformed by MLV or MSV, using a xenogeneic cytotoxic antibody assay. The identification of p30 in leukaemic mouse sera and the relevance of circulating p30 to the tumour status of the animal are discussed in the following paper by Epstein and Knight (1975). The results obtained here confirm and extend those previously reported with MLV-G induced lymphomata in the rat (Knight, Mitchison and Shellam, 1975).

### MATERIALS AND METHODS

*Mice.*—3–4 week old nonspecific pathogen-free female BALB/c mice were obtained from the ICRF breeding unit at Mill Hill. They were employed in these studies at 5–7 weeks of age after evidence was observed of successful intradermal scarification with reconstituted Lister Institute smallpox vaccine.

### *Tumours*

1. *Moloney leukaemia.*—Spleen cells from BALB/c mice infected with MLV-M were obtained from Dr Robert Seeger and injected intraperitoneally (i.p.) into newborn BALB/c mice. Thereafter the tumour was passaged in BALB/c mice at weekly intervals by i.p. injection of $10^7$ viable leukaemic spleen cells. For use as targets in the cytotoxic assay, erythrocytes in leukaemic spleens were lysed by Tris buffered ammonium chloride as described by Boyle (1968). $^{51}Cr$ labelling of spleen cells taken 7, 8, 12 or 19 days after passage of the leukaemia was performed in Hepes buffered MEM with 10% FCS as described previously (Knight *et al.*, 1975).

2. *MSC line.*—MSC is the designation for an *in vitro* line of cells developed at NIH and derived from an MSV-M induced sarcoma from BALB/c mice (Massicot, Woods and Chirigos, 1971). It was obtained for these studies from Dr Robert Seeger who originally obtained the line from Dr M. A. Chirigos at NIH. Both round and fibroblastoid types of cells are seen in MSC cultures. MSC is a viral transformed line as evidenced by loss of contact inhibition and its ability to form colonies in soft agar (Massicot *et al.*, 1971). Large amounts of MSV-M are released from the cultures. The cells were maintained in 25 ml Falcon plastic tissue culture flasks in E-4-G1 with 10% heat inactivated FCS in a $CO_2$ incubator and passaged every 3–4 days. For use in the cytotoxic assay, cells were removed from the flasks by treatment at 37°C with 0·02% versene in pH 7·2 phosphate buffered NaCl (PBS) pH 7·2, washed twice with warm Earle's saline, and then resuspended at $1 \times 10^7$ viable cells/ml in Hepes buffered MEM with 10% FCS in preparation for $^{51}Cr$ labelling.

### *Complement*

Rabbit complement was prepared as described previously (Knight *et al.*, 1975), and diluted 1 : 3, making the final dilution in the assay of 1 : 9.

*Antisera*

1. *Rat ILR-3.*—This antiserum was prepared in W/Fu rats against syngeneic MLV-M induced-lymphoma cells as described previously (Knight *et al.*, 1975). On Ouchterlony gel diffusion a precipitin line was formed with this antiserum and p30, and with disrupted MLV-M, but not with hamster sarcoma virus (HSV).

2. *Goat Anti-gs$_3$.*—This antiserum was prepared in goats against a preparation of ether disrupted FeLV. It was a gift from Professor William Jarrett, University of Glasgow, Glasgow. Additional details of its preparation and characterization have been described previously (Jarrett *et al.*, 1973; Knight, Mitchison and Shellam, 1975). This antiserum gave a line of identity not only with ether disrupted FeLV, but also with similar preparations of HSV and MLV on Ouchterlony gel diffusion. Hence it is primarily directed against gs$_3$ or the interspecies determinant of p30.

3. *Rat anti-formol MSV-M.*—MSV-M was treated for 2 weeks at 5°C with 0·1% formaldehyde and then dialysed exhaustively against phosphate buffered saline for 1 week. 50 μg of the formalinized virus was injected i.p. into adult W/Fu rats every week for 3 weeks and the animals were bled 10 days after the last injection. This antiserum is virus neutralizing and therefore recognizes the viral envelope antigen (VEA). It does not react with purified p30 on Ouchterlony gel diffusion.

4. *Moloney sarcoma virus regressor serum.*—This serum was obtained from Dr Robert Seeger. It was obtained from BALB/c mice 4 weeks after regression of a MSV-M induced sarcoma. Additional sera were prepared in BALB/c mice against MLV-M induced leukaemias from C3H mice and absorbed with normal C3H lymphoid tissue. All antisera were heat inactivated at 56°C for 30 min and stored in small aliquots at −20° before use in the cytotoxicity assay.

*Materials used for absorption of antisera*

1. *Viruses.*—Intact MSV, of the Moloney (M) or Gross (G) pseudo-type and intact MLV-G were propagated in tissue culture, purified by differential centrifugation and isopycnic centrifugation in sucrose and then dialysed against PBS as described previously (Knight and Gorczynski, 1975). Because of sensitivity of viral particles to freezing and thawing, it is possible that intact virus preparations also contain some internal virion proteins. Newcastle Disease virus (NDV) was a gift from Dr J. Skehel, National Institute of Medical Research, Mill Hill, London, England. Disrupted virus preparations were prepared either by 12 cycles of repeated freezing and thawing, or by Triton X-100 treatment followed by partition in ether as previously described (Knight and Gorczynski, 1975). The amount of protein was determined by the method of Lowry *et al.* (1951), or by the ratio of optical density at 280/260 nm using u.v.

2. *p30.*—p30 was prepared by an isoelectric focusing method described previously (Scolnick, Parks and Livingston, 1972). The purified material was at least 97% pure as judged by polyacrylamide gel electrophoresis. The mean molecular weight of 5 such preparations, estimated from gel electrophoresis was 32,500.

3. *Extracts of viral infected cells.*—Cell extracts of MSV-M and MSV-G infected lines of Theiler's original (To) mouse embryo fibroblasts were prepared by the hypertonic KCl technique described by Meltzer *et al.*(1971).

4. *Leukaemic and normal BALB/c spleen cell preparations.*—Single cell suspensions were prepared from pools of 2–3 leukaemic or normal animals, by teasing, filtration through stainless steel mesh and twice washing with E-4-G1. The cells were packed at 1500 rev/min for 10 min for use in absorption of antisera.

5. *Method of absorption.*—Varying amounts of the absorbents, *i.e.* either intact or disrupted virus, purified viral antigens, or KCl extracts of viral infected cells, were combined with the undiluted antisera to be absorbed, and sufficient E-4-G1 with 10% normal serum to make a final volume of 0·1 ml. This would result in the dilution of the antiserum desired for use in the cytotoxicity assay. Consequently, in most instances, 10–20 μl of antiserum were used, 10–70 μl absorbent, and the remainder was E-4-G1. The mixtures were maintained at room temperature for 30 min and then used in the cytotoxicity assays. Control preparations containing only the absorbents and on antisera were also included to exclude any possible cytotoxic or anticomplementary effects of the absorbents.

When antisera were absorbed with normal or leukaemic spleen cells, in some instances 250 $\mu$l of 1/5 dilutions of antisera were combined with 50 $\mu$l packed cells and incubated at 37°C for 30 min on a rotator. The cells were then pelleted at 2000 rev/min for 4 min. The procedure was repeated for a second absorption. On other occasions a fixed number of leukaemic or normal spleen cells was employed for the absorptions.

*Cytotoxicity assay*

1. *$^{51}$Cr labelling*.—The technique employed was that described by Knight *et al.*, (1975) with the following modification. $1 \times 10^8$ viable spleen cells from Moloney leukaemia bearing mice, or $1 \times 10^7$ viable MSC cells were suspended in 1 ml of Hepes buffered MEM with 10% heat inactivated FCS in 30 mm plastic Petri dishes and incubated with 100 $\mu$Ci of $^{51}$Cr (as Na chromate in aqueous solution: specific activity of 250 $\mu$Ci/$\mu$g, The Radiochemical Centre, Amersham) at 37°C for 1 h with gentle agitation on a rocking platform. The cells were spun at 1000 rev/min and washed 3 times with 40 ml of fresh media. The labelled spleen cells were resuspended at $5 \times 10^6$ viable cells/ml and the labelled MSC cells at $1 \times 10^6$ viable cells/ml.

2. *Assay*.—The assay was performed in $2 \times \frac{3}{8}$ in glass tubes (United Glass London), as described previously (Knight *et al.*, 1975). Serial dilutions (0·1 ml) of antisera to be tested were made in E-4-G1 media containing 10% heat inactivated normal serum of the same species from which the antiserum was derived. To this was added either 0·1 ml of the $^{51}$Cr labelled spleen cells ($5 \times 10^5$) or MSC cells ($1 \times 10^5$), and 0·1 ml rabbit complement diluted 1 : 3 with E-4-G1. The following controls were included in each assay: (1) $^{51}$Cr labelled cells and 0·2 ml E-4-G1 to determine total amount of label and amount of spontaneous release; (2) $^{51}$Cr labelled cells plus 0·2 ml 5% Brij 35 (polyoxyethylene lauryl ether, BDH Chemicals Ltd, Poole, England) to determine maximum release of label by detergent release; (3) $^{51}$Cr labelled cells plus rabbit complement and 10% normal serum in E-4-G1, without antiserum, to determine the extent of the background cytotoxicity of the complement itself; (4) $^{51}$Cr labelled cells plus either rabbit complement or 10% normal sera without antisera to study the selective effect of either the complement or the diluent used for antisera on the target cells.

The tubes were incubated for 45 min at 37°C in a 5% $CO_2$ atmosphere. After incubation, 1 ml of cold Earle's saline was added to each tube and all tubes except those under study for total $^{51}$Cr labelling were centrifuged at 5°C for 5 min at 2000 rev/min. Supernatants were decanted and counted on a Wallac Gamma Counter (Model GTL 300, Wallac, Turku, Finland).

In some instances a 2-step assay was performed, in which cells and dilutions of antisera being absorbed with various substances were incubated alone at 37°C for 30 min before the addition of rabbit complement and then incubated for an additional 30 min at 37°. Such samples were run in this manner to minimize any anti-complementary effects of the absorbents.

For these studies % specific cytotoxicity was defined as follows:

$$\frac{\%\text{ specific}}{\text{cytotoxicity}} = \frac{\text{ct/min}_{\text{Ab+C}} - \text{ct/min}_{\text{NS+C}}}{\text{ct/min}_{\text{Brij}} - \text{ct/min}_{\text{NS+C}}}$$

where ct/min = Counts per min in tubes containing the following substances:
Ab = Various dilutions of a given antiserum
C = rabbit complement
NS = 10% normal serum, used as diluent
Brij = detergent, *i.e.* maximum release of counts.

To determine the extent to which a given substance will absorb antibody activity, *i.e.* block complement dependent antibody mediated cytotoxicity, the following formula was employed.

$$\%\text{ block} = \frac{\%\text{ specific cytotoxicity}_{\text{Ab}} - \%\text{ specific cytotoxicity}_{\text{Ab+Y}}}{\%\text{ specific cytotoxicity}_{\text{Ab}}} \times 100$$

where

Ab = Various dilutions of a given antiserum
Ab + Y = various dilutions of a given antiserum in the presence of absorbent, Y.

### RESULTS

*Complement dependent cytotoxicity of Rat ILR-3*

The graph in the left portion of Fig. 1 depicts the results of 4 experiments which show that Rat ILR-3 serum contains antibodies which are cytotoxic for M leukaemic spleen cell targets in the

FIG. 1.—Complement dependent % specific cytotoxicity of Rat ILR-3 for M leukaemia spleen cell and MSC targets. The symbols indicate either days after passage of leukaemia *in vivo* or passage of cells *in vitro*.

For experiments on M targets, ● and ○ were from the same group of animals studied 7 and 12 days after passage of leukaemia; □ and ■ were studied on the same day, but each originated from a separate group of animals, distinct from that depicted with ● and ○. For experiments on MSC targets, ●, ○ and □ were studied sequentially, from the same passage. ■ indicates a separate passage, distinct from □.

A negative value indicates that there was less $^{51}Cr$ release than in the control tubes containing 10% normal rat serum and rabbit complement, but still more than the spontaneous release.

presence of rabbit complement. Preliminary experiments had shown that greater % specific cytotoxicity was achieved with this antiserum and rabbit complement than with guinea-pig complement. The % cytotoxicity of Rat ILR-3 for M targets observed in these experiments are in good agreement with similar experiments performed by Knight *et al.* (1975). For a given experiment the mean difference between the calculated % specific cytotoxicity of duplicate samples was 6%. In no instance was the difference between the replicates greater than 15%.

If the data from all 4 experiments are pooled, of the total $^{51}Cr$ label, $20 \pm 3{\cdot}2\%$ of the counts were released spontaneously, $62 \pm 5{\cdot}0\%$ were released by detergent, and $35 \pm 4{\cdot}6\%$ were released by 10% normal rat serum and rabbit complement. Slight variation in response was observed from one group of animals to another, but in general these and other similar experiments suggested that for a given group of animals the % specific cytotoxicity at lower dilutions of Rat ILR-3 was greater with 7 and 12 day targets than with 19 day cells.

The data depicted on the right side of

FIG. 2.—Complement dependent % specific cytotoxicity of Rat ILR-3 for M leukaemia spleen cell targets before and after absorption with either M leukaemia spleen cells or normal spleen cells from BALB/c mice. ● non absorbed, △ absorbed × 1 with normal cells, ▲ absorbed × 2 with normal cells, □ absorbed × 1 with M leukaemia cells, ■ absorbed × 2 with M leukaemia cells.
Leukaemia cells used for absorptions were obtained 7 days after passage of leukaemia and absorptions were carried out as detailed in Materials and Methods. A negative value indicates that there was less $^{51}$Cr release than in the control tubes containing 10% normal rat serum and rabbit complement, but still more than the spontaneous release.

Fig. 1 are from 4 representative experiments and demonstrate that ILR-3 contains antibodies which are also cytotoxic for MSC targets in the presence of rabbit complement. With these target cells, of the total $^{51}$Cr label, spontaneous release amounted to 11 ± 5·4%, detergent release 65 ± 6·5% (with the exception of cells studied one day after passage, where detergent release accounted for only 18% of the total $^{51}$Cr label), and 10% normal rat serum and rabbit complement accounted for 14 ± 1·6% (again with exception of cells studied one day after passage, where the value was only 4%).

*Absorption studies with Rat ILR-3*

To identify the antigenic determinants which served as targets for the complement dependent cytotoxic antibodies in Rat ILR-3, several absorption studies were performed. Figure 2 depicts the % specific cytotoxicity of Rat ILR-3 for M leukaemic spleen cell targets before and after one and 2 absorptions with either normal spleen cells or M leukaemic spleen cells obtained from BALB/c mice. The data indicate that M leukaemic spleen cells did absorb the cytotoxic antibody activity, partially by the first and completely by the second absorption. In

FIG. 3.—% block of antibody mediated cytotoxicity of Rat ILR-3 for M leukaemia spleen cell targets by intact and disrupted MSV-M virus. ● intact MSV-M, ○ disrupted MSV-M.
Values observed above 100% block indicate that the absorbents had protective effect not only against cytotoxicity of the antiserum tested, but also against the cytotoxicity imparted by rabbit complement as well. Leukaemic cells obtained 12 days after passage of leukaemia employed in these studies.

contrast, normal BALB/c spleen cells were ineffective in absorbing the cytotoxic antibody, even after 2 absorptions. This indicates, therefore, that the cytotoxic antibody or antibodies present in ILR-3 are directed to components on the surface of M leukaemic spleen cells that are distinct from that seen on normal spleen cells, and which are therefore related to leukaemic associated antigens.

Subsequently, absorption studies on Rat ILR-3 were performed with intact and disrupted MSV-M virus preparations. The data from a representative experiment using M leukaemic spleen cell targets are shown in Fig. 3 and indicate that both intact and disrupted viral preparations can absorb cytotoxic antibodies from Rat ILR-3. As little as 28 µg protein of disrupted virus and 35 µg protein of intact virus block 100% of the reaction. This suggests that ILR-3 contains cytotoxic antibodies against both internal and external virion antigens.

To determine the specificity of the viral block, virus unrelated to the C type RNA viruses was also employed in similar Rat ILR-3 absorption studies, but with MSC cells as targets. One representative experiment is depicted in Fig. 4. It is apparent that for MSC targets as well as for M leukaemic spleen targets, that only a small amount of MSV-M protein (10 µg) can result in greater than 90% block of the cytotoxic activity of ILR-3. Weight for weight, MSV-M was 1000 times as efficient in blocking as NDV. Extrapolation of the data obtained with NDV indicates that $>$ 10 mg of NDV would be required to produce inhibition equivalent to that produced by 10 µg of MSV-M.

The block of cytotoxic activity of ILR-3 for MSC targets was not unique for MSV-M, however. Other related C type RNA viruses, MSV-G and MLV-G, as well as KCl extracts of mouse embryo tissue culture lines infected with MSV-G and MSV-M, could absorb the cytotoxic antibodies in Rat ILR-3, and the data are depicted in Fig. 5. In these experiments,

FIG. 4.—% block of antibody mediated cytotoxicity of Rat ILR-3 for MSC targets by intact MSV-M and NDV virus.

FIG. 5.—% block of antibody mediated cytotoxicity of Rat ILR-3 for MSC targets by intact MSV-M, MSV-G, MLV-G and KCl extracts of mouse embryo tissue culture lines infected with MSV-G or MSV-M.

100% block was obtained with 8 µg of MSV-M or MSV-G and < 12 µg MLV-G. If the data obtained using KCl extracts are extrapolated to 100%, 160 µg of MSV-M infected and 240 µg of MSV-G infected preparations were required. Therefore, weight for weight, 20–30 times as much KCl extract of virus infected cells were required for 100% block, than intact virus.

*Cytotoxicity studies with monospecific antisera*

To confirm the presence of specific viral antigens on the surface of the M leukaemia spleen cell and MSC targets, monospecific antisera were employed. Figure 6 depicts the results of 2 experiments in which the % specific cytotoxicity of rat anti-formol MSV-M serum for M leukaemia spleen cell targets was studied.

FIG. 6.—Complement dependent % specific cytotoxicity of rat anti-formol MSV-M for M leukaemia spleen cell targets 7 and 12 days after passage of leukaemia.

FIG. 7.—Complement dependent % specific cytotoxicity of goat anti-$gs_3$ for MSC targets.

FIG. 8.—Complement dependent % specific cytotoxicity of goat anti-$gs_3$ for M leukaemia spleen cell targets before and after absorption with normal or leukaemic spleen cells. ● non-absorbed, ○ absorbed with the number of cells indicated. The first 3 panels represent absorptions with leukaemic cells taken after passage of the leukaemia at the time indicated. The far right panel represents the absorption with normal spleen cells.

In the presence of rabbit complement, cytotoxicity was observed against cells obtained 7 and 12 days after passage of the leukaemia, but higher dilutions of the antiserum were more cytotoxic for the Day 7 cells than for the Day 12 cells.

In subsequent experiments, antisera directed against internal virion proteins were studied. Figure 7 illustrates a representative experiment in which goat anti-$gs_3$ was shown to be cytotoxic for MSC target cells in the presence of rabbit complement. Goat anti-$gs_3$ was also cytotoxic for M leukaemia spleen cell targets, although to a lesser degree than was observed with MSC targets. For example, in 4 experiments with M targets the mean % cytotoxicity at a dilution of 1/5 was 16%; at 1/10, 13%; at 1/20, 11%, at 1/40, 1%. A comparison was then made between the % specific cytotoxicity observed before and after absorption of the goat anti-$gs_3$ with either normal BALB/c spleen cells or M leukaemia spleen cells, the latter obtained at 4, 8 and 12 days after the passage of the leukaemia. The results, shown in Fig. 8, indicate that spleen cells obtained from the leukaemic animals could absorb out the anti-$gs_3$ cytotoxic antibodies, but that normal spleen cells could not. It was of interest to note that spleen cells obtained 8 days after the passage of leukaemia were superior to those obtained either after 4 or 12 days, as in the former instance most of the anti $gs_3$ activity was absorbed with only $10^5$ cells, whereas in the latter 2 instances more cells were required.

### *Absorption studies with purified viral proteins*

Several experiments were performed to substantiate the role of p30 as a cyto-

TABLE.—*Effect of Absorption of Antisera with Viral Protein on % Specific Cytotoxicity*

| Antiserum | Dilution absorbed | Viral protein | Amount used for absorption (μg) | % Specific before absorption | Cytotoxicity* after absorption | % Block |
|---|---|---|---|---|---|---|
| MSC targets | | | | | | |
| Rat ILR-3 | 1/10 | p30 | 0·18 | 42† | 24 | 43 |
| | | | 1·8 | 42 | 17 | 59 |
| | | | 3·6 | 42 | 15 | 64 |
| | | | 9·0 | 42 | 11 | 74 |
| | | | 12·6 | 42 | 7 | 83 |
| M leukaemia targets | | | | | | |
| Rat ILR-3 | 1/5 | p30 | 3·1 | 69 | 58 | 16 |
| Rat anti-formol MSV-M | 1/20 | p30 | 3·6 | 15 | 18 | 0 |

* % specific cytotoxicity and % block are calculated as described in Materials and Methods.

† Values expressed are the means of duplicate determinations. In these experiments the mean difference between the calculated % specific cytotoxicity of duplicate samples was 5 ± 3%. In no instance was the difference between the replicates greater than 8%.

toxic target on MSC and M leukaemia spleen cell targets and they are summarized in the Table. Absorption of Rat ILR-3 with as little as 0·18 μg of p30 resulted in 43% block of the cytotoxic activity of the antiserum. Yet, increasing the amount of p30 used for the absorption by 70-fold, *i.e.* to 12·6 μg still did not result in 100% absorption of cytotoxic activity.

Absorption of ILR-3 antibody with 3·6 μg p30 produced 64% block of cytotoxicity against MSC targets. However, the same amount of antigen inhibited lysis of M leukaemia spleen cell targets by ILR-3 by only 16%. On both cell targets ILR-3 is recognizing p30, although it would appear that even on MSC cells its cytotoxicity is not directed exclusively to this antigen.

The goat anti-$gs_3$ antiserum was cytotoxic for both MSC and M leukaemia cell targets, although the % specific cytotoxicity of leukaemic cells was low. Correspondingly, as little as 0·18 μg purified p30 totally absorbed cytotoxicity against leukaemic cells, whereas only 20% of the cytotoxicity against MSC cells was absorbed by 9·0 μg purified p30.

p30 was not effective in absorbing cytotoxic activity of rat anti-formol MSV-M, suggesting that the absorption of other antisera by p30 is a specific event. The cytotoxicity of the rat anti-formol MSV-M confirms the presence of cytotoxic targets other than p30 on the surface of leukaemic cells.

To rule out further the possibility of nonspecific absorption, *i.e.* that any protein, if in large enough amounts could absorb out cytotoxic antibody activity, a parallel experiment using Rat ILR-3 (absorbed with amounts of albumen varying from 30 μg to 6 mg) against MSC targets was run. Absorption by albumen was noted only with 6 mg, an amount far in excess of the amount of viral or cellular protein used in our other blocking studies.

*Cytotoxicity studies with MSV regressor sera*

In 4 experiments using MSC targets, with either guinea-pig complement or rabbit complement, and using 2 separate BALB/c MSV regressor sera neat, and in doubling dilutions to 1/640, no cytotoxic activity was noted. Similar negative findings were observed when M leukaemia spleen cells were employed as targets with the antiserum prepared in BALB/c mice against $C_3H$ MLV-M induced leukaemia. This result was not unexpected as mouse sera are thought not to recognize p30.

DISCUSSION

Spleen cells from mice bearing Moloney leukaemia were chosen for study in these experiments because they represent cells infected by C type RNA viruses taken from an *in vivo* environment in which host defence mechanisms come into play. By contrast, MSC cells, also chosen for study, represent a cell type or types transformed by such viruses, and taken from an *in vitro* environment without benefit of host defence mechanisms other than those inherent in the cells themselves.

The present study demonstrates that p30, the most abundant internal virion protein of C type RNA viruses, is associated with the surface on both these cell types, in that group specific antigenic determinants on the p30 molecule were shown to act as cytotoxic targets in xenogeneic complement dependent antibody mediated cytotoxicity reactions.

For this study we employed an antiserum prepared in rats against a syngeneic MLV-M lymphoma (ILR-3) and one prepared in goats against an ether disrupted preparation of FeLV (goat anti-$gs_3$). Absorption of each of these antisera with normal BALB/c mouse spleen cells resulted in no loss of cytotoxic activity, thus indicating that the cytotoxic antibodies present in these sera were directed to antigenic determinants other than those found on normal murine cells. This observation, coupled with the fact that absorption of each antiserum with Moloney leukaemia spleen cells from age and sex matched BALB/c mice resulted in complete loss of cytotoxic antibody activity, indicated that such antibodies were directed toward leukaemia associated antigens.

Further absorption studies with Rat ILR-3 revealed the following information about the cytotoxic antibodies contained therein: (1) They were directed against antigenic determinants associated with oncogenic C type RNA viruses, but not with non-oncogenic, unrelated RNA viruses as significant absorption of cytotoxic antibody activity against MSC cells was observed with intact MSV-M but not with NDV. (2) They were directed against antigenic determinants of internal as well as external proteins of MSV-M, as both disrupted, as well as intact viral preparations of MSV-M absorbed cytotoxic activity directed against M leukaemia spleen cell targets. (3) They were directed against group specific antigenic determinants of the C type RNA viruses as intact MSV-G and MLV-G as well as MSV-M absorbed cytotoxic activity directed against MSC targets. (4) They were not heteroantibodies directed against murine histocompatibility antigens (which have been shown on occasion to adhere to viral envelopes (Aoki and Takahashi, 1972), as MLV-G propagated in rat tissue culture lines absorbed the cytotoxic activity of ILR-3 as effectively as MSV-G and MSV-M propagated in mouse tissue culture lines. (5) They were directed against group specific viral antigenic determinants, which were intimately associated with the surface membranes of cells infected with C type RNA viruses, as KCl extracts of whole mouse embryo cells infected with either MSV-M or MSV-G absorbed cytotoxic antibody activity of ILR-3 against MSC targets. (6) They were directed against antigenic determinants of p30 obtained from MSV-M as highly purified preparations of this protein were effective in absorbing cytotoxic antibody activity of ILR-3 against both M leukaemia spleen cell and MSC targets.

Confirmatory evidence that p30 is associated with the surface membranes of both cell types was obtained with the goat anti-$gs_3$ serum. It was cytotoxic for both cell types and its cytotoxic antibody activity could be partially blocked by purified preparations of p30. It is possible that the antigenic determinants of p30 are more abundant or more accessible on the surface of MSC cells than on M leukaemia target cells, as there was more specific cytotoxicity for the former than the latter. Also, a given amount of p30 blocked the cytotoxicity of this antiserum for M targets more readily than MSC, despite the fact that 5 times as many M targets were employed as MSC. There is also suggestive evidence that the amount or accessibility of the antigenic determinants of p30 on M target cells can fluctuate, as a fixed number of leukaemic cells obtained 8 days after inception of the leukaemia were more effective at absorbing the cytotoxic activity of the goat anti-$gs_3$ than those obtained either at Day 4 or Day 12. However, in this situation it is difficult to distinguish between the presence of more $gs_3$ per cell or simply the presence of more cells with $gs_3$. It is possible that the quantitative differences between the expression of p30 antigenic determinants on MSC and M cells, or on M cells with time are due to host modification of antigen expression on the *in vivo* targets.

It is unlikely that the presence of p30 on viral infected or transformed cells represents simple adsorption of the viral protein to the surface membranes of cells. In the present study, target cells were washed 4–5 times before use in the cytotoxic assay and still the antigenic determinants of p30 were detectable as cytotoxic targets. Furthermore, Yoshiki *et al.* (1973, 1974) have demonstrated with immunoelectron microscopic techniques that p30 is located on cell membranes near the site of virus budding but at a locations distinct from that of VEA. They then showed with immunofluorescence that an antigenic determinant of p30 can actually cap with monospecific antisera, thus indicating an intimate relationship of the p30 molecule with the fluid membrane of C-type RNA virus infected cells.

Despite the fact that considerable cytotoxicity was observed in the present study with the goat anti-$gs_3$ for MSC cells, we were unable to produce more than 14% specific lysis of MSC cells or any lysis of M leukaemia spleen cells with a rabbit antibody raised to purified p30 and which recognized solely the $gs_1$ determinant.

It would appear then that in the mouse differential expression of $gs_1$ and $gs_3$ antigenic determinants of p30 occurs. This is not true for the rat, where the same goat anti-$gs_3$ and rabbit anti-$gs_1$ sera employed in the present study gave equal amounts of cytotoxicity when tested against rat G lymphoma cells (Knight *et al.*, 1975).

The question still remains as to whether Rat ILR-3 and goat anti-$gs_3$ have cytotoxic antibodies to antigenic determinants other than to those detected on the p30 molecule. With the doses of p30 used in this study, up to 83% of the cytotoxic activity of ILR-3 against MSC targets was absorbed but only 16% of the activity against M targets was observed. There is no doubt that other leukaemia associated antigens are present on the surface of both target cell types. Rat anti-formol MSV-M serum was cytotoxic for the MSC cells and its activity, directed primarily against VEA, could not be blocked by p30. Similarly, a minimal amount of block (10%) was observed where ILR-3 was absorbed with 2 $\mu$g of pure VEA (Epstein, Mitchison and Knight, unpublished). In addition, intact MSV-M was shown to absorb ILR-3 almost equally as well as disrupted MSV-M. The disrupted virus preparations contain approximately 100 times as much p30 as intact virus. If the antigenic determinants of p30 were the sole specificities recognized by ILR-3, one would have expected better absorption with the disrupted virus preparations.

Recently, Ferrer suggested that GCSA (b), a serologically detected antigenic determinant on the surface of Gross virus induced lymphomata which is detected by rat antisera but not by mouse, is p30 (Ferrer, 1973).

The results of the present study are in agreement with this, as are our recent studies which showed total block of the cytotoxicity of rat anti-Gross lymphoma sera for syngeneic target cells by p30 (Knight and Gorczynski, 1975; Knight *et al.*, 1975). A manuscript to follow also offers confirmatory evidence, as in it we demonstrate that Moloney leukaemic serum contains p30 and also absorbs the cytotoxic activity of Rat ILR-3 against MSC and M target cells (Epstein and Knight, 1975).

We gratefully acknowledge the expert technical help of Jenny Bruce and Keith Adams. We thank Professor N. A. Mitchison for his advice and encouragement. We are indebted to Carol Stadum and Dorothy Metcalf for typing the final manuscript.

### REFERENCES

AOKI, T. & TAKAHASHI, T. (1972) Viral and Cellular Surface Antigens of Murine Leukemias and Myelomas. Serological Analysis by Immunoelectron Microscopy. *J. exp. Med.*, **135**, 443.

BOLOGNESI, D. P., LUFTIG, R. & SHAPER, J. H. (1973) Localization of RNA Tumor Virus Polypeptides. *Virology*, **56**, 549.

BOYLE, W. (1968) An Extension of the Cr-Release Assay for the Estimation of Mouse Cytotoxins. *Transplantation*, **6**, 761.

EPSTEIN, L. B. & KNIGHT, R. A. (1975) Studies on Mouse Moloney Virus Induced Tumours. II. Detection of p30 in the Serum of Mice with Moloney Leukaemia by *in vitro* Blocking of Complement Dependent Antibody Mediated Cytotoxicity. *Br. J. Cancer*, **31**, 513.

FERRER, J. F. (1973) Cell-surface and Virion-envelope Antigens Shared by Radiation Leukemia Virus (Rad LV) and other Murine C-type Viruses. *Int. J. Cancer*, **12**, 378.

GEERING, G., OLD, L. J. & BOYSE, E. A. (1966) Antigens of Leukemias Induced by Naturally Occurring Murine Leukemia Virus: Their Relation to the Antigens of Gross Virus and other Murine Leukemia Viruses. *J. exp. Med.*, **124**, 753.

GILDEN, R. V., OROSZLAN, S. & HUEBNER, R. J. (1971) Coexistence of Intraspecies and Interspecies Specific Antigenic Determinants on the Major Structural Polypeptide of Mammalian C-type Viruses. *Nature, New Biol.*, **231**, 107.

GORCZYNSKI, R. M. & KNIGHT, R. A. (1975*a*) Cell Mediated Immunity to Moloney Sarcoma Virus in Mice. IV. Direct Cellular Cytolysis of $^{51}$Cr Labelled Target Cells *in vitro* and Analysis of Blocking Factors which Modulate Cytotoxicity. *Br. J. Cancer*, **31**, 387.

GORCZYNSKI, R. M. & KNIGHT, R. A. (1975*b*) Cell-mediated Immunity to Moloney Sarcoma Virus in Mice. II. Analysis of Antigenic Specificities Involved in T-lymphocyte-mediated *in vivo* Rejection of Murine Sarcoma Virus-induced Tumors. *Eur. J. Immunol.* In the press.

JARRETT, W. F. H., JARRETT, O., MACKEY, L., LAIRD, H. M., HARDY, W. D. Jr. & ESSEX, M. (1973) Horizontal Transmission of Leukemia Virus and Leukemia in the Cat. *J. natn. Cancer Inst.*, **51**, 833.

KNIGHT, R. A. & GORCZYNSKI, R. M. (1975) Cell Mediated Immunity to Moloney Sarcoma Virus in Mice. I. Analysis of Antigens Responsible for Lymphocyte Stimulation in Regressor Mice. *Int. J. Cancer*. **15**, 48.,

KNIGHT, R. A., MITCHISON, N. A. & SHELLAM, G. R. (1975) Studies on a Gross Virus-induced Lymphoma on the Rat. II. The Role of Cell Membrane Associated and Serum p30 Antigen in the Antibody and Cell-mediated Response. *Int. J. Cancer*. In the press.

LOWRY, O. H., ROSEBROUGH, N. J., FARR, A. L. & RANDALL, R. J. (1951) Protein Measurement with the Folin Phenol Reagent. *J. biol. Chem.*, **193**, 265.

MASSICOT, J. G., WOODS, W. A. & CHIRIGOS, M. A. (1971) Cell Line Derived from a Murine Sarcoma Virus (Moloney Pseudotype)-induced Tumor: Cultural, Antigenic and Virological Properties. *Appl. Microbiol.*, **22**, 1119.

MELTZER, M. S., LEONARD, E. J., RAPP, H. J. & BORSOS, T. (1971) Tumor-specific Antigen Solubilized by Hypertonic Potassium Chloride. *J. natn. Cancer Inst.*, **47**, 703.

PARKS, W. P. & SCOLNICK, E. M. (1972) Radioimmuno Assay of Mammalian Type-C Viral Proteins: Interspecies Antigenic Reactivities of the Major Internal Polypeptide. *Proc. natn. Acad. Sci. U.S.A.*, **69**, 1766.

SCOLNICK, E. M., PARKS, W. P. & LIVINGSTON, D. M. (1972) Radioimmunoassay of Mammalian Type C Viral Proteins. *J. Immun.*, **109**, 570.

STRAND, M. & AUGUST, J. T. (1974) Structural Proteins of Mammalian Oncogenic RNA Viruses; Multiple Antigenic Determinants of the Major Internal Protein and Envelope Glycoprotein. *J. Virol.*, **13**, 171.

YOSHIKI, T., MELLORS, R. C. & HARDY, W. D. JR (1973) Common Cell-surface Antigen Associated with Murine and Feline C-type RNA Leukemia Viruses. *Proc. natn. Acad. Sci. U.S.A.*, **70**, 1878.

YOSHIKI, T., MELLORS, R. C., HARDY, W. D. JR & FLEISSNER, E. (1974) Common Cell-surface Antigen Associated with Mammalian C-type RNA Viruses. *J. exp. Med.*, **139**, 925.